# Interrogating Raisin Associated Unsaturated Fatty Acid Derived Volatile Compounds Using HS–SPME with GC–MS

**DOI:** 10.3390/foods12030428

**Published:** 2023-01-17

**Authors:** Hafiz Umer Javed, Dong Wang, Abdullah, Murtaza Hasan, Li-Yan Zeng, Yibin Lan, Ying Shi, Chang-Qing Duan

**Affiliations:** 1 Center for Viticulture & Enology, College of Food Science and Nutritional Engineering, China Agricultural University, Beijing 100083, China; 2 Key Laboratory of Viticulture and Enology, Ministry of Agriculture, Beijing 100083, China; 3 School of Chemistry and Chemical Engineering, Zhongkai University of Agriculture and Engineering, Guangzhou 510225, China; 4 College of Food Sciences, South China Agricultural University, Guangzhou 510642, China

**Keywords:** Thompson seedless, sun-drying, model reaction, UFAO-derived compounds

## Abstract

This study proposed to investigate the generation mechanism of raisins-derived volatile compounds during unsaturated fatty acids oxidation (UFAO) using a mixture of fatty acids (FAs) and four individual FA at different time intervals (0, 4, 8, 12, 16, and 20 days; 60 °C). During the sun-drying of ‘Thompson Seedless’ grapes (*Vitis vinifera* L.), a total of 39 UFAO-derived volatiles were characterized by the GC-MS. Firstly a pH value of 4.2 was optimized to proceed with a raisin drying-like UFAO model reaction. Afterward, GC-MS quantification revealed 45 UFAO-derived volatiles, and the maximum numbers of compounds were identified in the interaction of all FAs (39) following linoleic acid (29), erucic acid (27), oleic acid (25), and linolenic acid (27). Pentanoic acid, (*E,E*)-2,4-octadienal, and *n*-decanoic acid were only quantified in all FAs, linoleic acid, and erucic acid, respectively. This study showed that all FAs reactions were found to be responsible for the generation of a greater number of UFAO-derived volatiles with higher concentrations.

## 1. Introduction

Conventional drying methods (sun and air) and modern drying techniques (microwave heating, freeze-drying, and oven-drying) are being used to produce raisins from fully ripened grapes [1]. According to the USDA (United State Department of Agriculture), China produces 1.90 Mt of raisins annually and contributes significantly to the global economy [2]. In the year 2016–17, China was placed third after USA and Turkey in terms of raisin production [3]. The main advantage of drying is responsible for the inhibition of microbial growth (e.g., molds, yeast, and bacteria). Moreover, the drying process also significantly affects the major physical (color, size, shape, and shrinkage) and biochemical (oxidation detrition and browning reaction) quality parameters [4]. Among the traditional methods, the sun-drying process is considered the most efficient, cost-competitive [4], and requires less time for making raisins [5,6]. Intense sun radiation and temperature differences are the key factors of the raisins’ color, appearance, and aroma [7]. In regards to the chemical composition of raisins, some volatile organic compounds (VOCs) such as 2-octanol, 1-butanol, and 3-methyl-2-buten-1-ol are produced by the sun-drying method [5], whereas several other VOCs (e.g., ethyl hexanoate, 2-pentyl furan, 2,6-diethyl pyrazine, β-damascenone, β-ionone) are formed through Maillard reaction (MR), unsaturated fatty acids oxidation (UFAO), glycosidically bound (GD), and carotenoids, resulting in different kinds of flavors such as fruity, floral, fatty, and roasted (Javed et al., 2019; Wang et al., 2017). Among these chemical reactions, UFAO is the main contributor to developing an attractive raisin aroma [5].

Furthermore, the lipid oxidation reaction especially UFAO is the major source for the generation of VOCs in raisins, and their product not only influences the consumer but also attracts the scientist in terms of the nutritional, sensory, and shelf life of the product. This process is initiated by the enzyme (lipoxygenase) and is carried out in plant tissues during growth, maturation, tissue disruption, fruit processing, storage, and transportation; it is responsible for flavor formation in vegetables and fruits [8]. The fatty acid-based compounds are the primary source of raisin aroma [5,9,10], comprising six- and nine-carbon compounds of alcohols, esters, and aldehydes produced by enzymatic breakdown, α- and β-oxidation of unsaturated fatty acids (UFAs) in intact or disrupted fruits [11]. Furthermore, these volatile are a primary source of aroma in fruits and wine formed by the oxidative degradation of UFAs [12]. This reaction starts via the autoxidation of UFAs [8] or the lipoxygenase (LOX) hydroperoxide lyase (HPL) pathway, in which the main enzymes such as LOX, HPL, and alcohol dehydrogenases (ADHs) contribute significantly to the synthesis of volatiles [13,14].

Raisins were reported to have more polyunsaturated fatty acids (PUFA) than saturated fatty acids (SFA) and monounsaturated fatty acids (MUFA). Palmitic acid (15.84–24.23%), oleic acid (9.72–16.01%), and linoleic acid (47.80–60.74%) were the most prevalent among SFA, MUFA, and PUFA, respectively [15]. In recent years, several studies reported that UFAs especially oleic acid, linoleic acid, and linolenic acid were the richest source of fatty acids in raisins [16,17] and were found responsible for the production of UFAO-derived volatiles by employing different traditional (sun and air) and chemical (fatty acid-based oil) drying techniques [5,6,9,10]. The compounds of aliphatic acids (decanoic acids, nonanoic acid, octanoic acid, heptanoic acid, and hexanoic acid) and aliphatic aldehydes (pentanal, heptanal, hexanal, (*E,E*)-2,4-nonadienal, (*E*)-2-nonanal, (*E*)-2-octenal, and (*E*)-2-heptenal) were identified in raisins with high concentrations [5], formed by the oxidation of oleic acid and linoleic acid [18,19], as well as producing green aroma in raisins [5,6,9,10].

In this study, the raisins drying model reaction was optimized to detect the origin of the fatty acid-derived compounds. To date, some research studies described that pH has a significant impact on the formation of UFAO-derived compounds in different meat-like model systems [20,21]. Likewise, some model studies were carried out at different temperatures [20,21,22,23] to understand the generation mechanism of fatty acids-derived compounds. However, no research has been conducted to determine how the raisin drying model system works to produce specific UFAO compounds. Therefore, the current study aimed to characterize and quantify the UFAO compounds in ‘Thompson Seedless’ during the sun-drying processing at various intervals (0, 4, 8, 12, 16, and 20 days). After that, the pH of the model reaction was tuned, and then a detailed investigation was made to determine the generation of UFAO-derived volatile compounds as well as their source.

## 2. Materials and Methods

‘Thompson Seedless’ grapes were obtained from the commercial orchard, which is located in the Xinjiang province of China. The grape berries were harvested when fully mature and with total soluble solids (TSS) above 20 Brix. Then, they were immediately subjected to traditional sun drying. The sun-drying process was continued for 20 days and did not stop until the water content (˂15%) of ‘Thompson Seedless’ remained constant for three days. The loss of moisture during the drying of ‘Thompson Seedless’ raisin was shown in Figure S1. The 1 kg samples for each replicate (*n* = 3) were collected on different days 0, 4, 8, 12, 16, and 20 of the drying process and transferred to the College of Food Science and Nutritional Engineering (FSNE) at China Agricultural University (CAU) in Beijing for the identification and quantification of volatile compounds. Before analysis, all the grape samples were quickly stored in a refrigerator (−40 °C).

### 2.1. Chemicals

For the oxidation of fatty acids, Sigma^®^-Aldrich provided erucic acid (C22:1), linolenic acid (C18:3), linoleic acid (C18:2), oleic acid (C18:1), and stearic acid (C18:0).

Tetrasodium pyrophosphate decahydrate (Na_4_O7P_2_·10H_2_O), sodium phosphate monobasic dihydrate (NaH_2_PO_4_·2H_2_O), hydrochloric acid (HCl), and sodium chloride (NaCl) were procured from Beijing chemical works (BCW), Beijing, China. The pure distilled water was obtained by a Milli-Q sanitization organization (Millipore, Bedford, MA, USA). Further, the standards were the identification and quantification of volatile compounds that were mentioned in Table S1.

### 2.2. Preparation of Free-Form Volatiles

The free-form volatiles were identified as stated in our prior research [24], with slight modifications. One hundred berries were separately chosen from fresh grapes and then the juice was collected by hand using polyethylene bags (food-grade). In the case of raisins, 100 berries were soaked overnight using an equivalent weight of distilled water. The very next day soaked samples were well mixed and macerated for 240 min, and then immediately centrifuged (8000 rpm; 4 °C for 10 min). All samples were immediately analyzed for the characterization of free-form volatiles.

### 2.3. Optimization of pH for Model Reaction

The buffer solution’s pH was initially optimized for the UFAO model reaction. For this purpose, pyrophosphate buffer (0.2 M) was prepared by mixing tetrasodium pyrophosphate decahydrate (Na_4_O_7_P_2_.10H_2_O) and sodium phosphate monobasic dihydrate (NaH_2_PO_4_.2H_2_O) in distilled water. The pH of the pyrophosphate buffer was configured at a level of 4.2, 6.2, and 7 with the help of NaOH (2 M) and HCl (2 M). In a 20 mL reaction tube, 0.20 mM of each fatty acid (stearic acid, oleic acid, linoleic acid, linolenic acid, erucic acid) was added and blended with the help of an electric blender. The sample tubes were then kept in a controlled oven at 60 °C for 10 and 20 days. After the removal from the oven, all samples were stored at −80 °C until they were analyzed by Gas Chromatography-Mass Spectrometry (GC-MS).

### 2.4. UFAO Model Reaction

**Figure 1:**
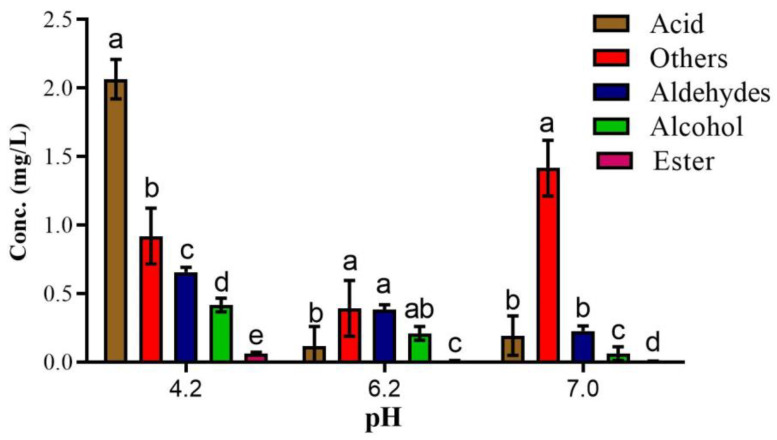
The concentration of identified UFAO-derived compounds in model reactions at different pH. Mean ± standard error (*n* = 3); Different lettering indicates a significance level *p* < 0.005.

The pH of the reaction was optimized based on pre-experiments (4.2) for the UFAO model reaction (Figure 1). According to a prior study, when ‘Thompson Seedless’ grapes were dried, the pH and temperature inside the berries were similar to the optimal pH value and selected temperature. [25].

Fatty acid oxidation reactions were carried out in pyrophosphate buffer at pH 4.2, with each reaction mixture containing 0.3 mL of fatty acids being blended in a 20 mL reaction tube using an electric blender. The following are the seven reaction mixtures: (1) control; (2) stearic acid; oleic acid (3); linoleic acid (4); linolenic acid (5); erucic acid (6) and (7) all fatty acids with the duplicate. After that, all the sample tubes were kept in a controlled oven at 60 °C for 20 days. The reaction samples were removed from the oven at an interval of 4 days, cooled at room temperature, and then kept in a refrigerator at −80 °C. Before analysis in GC-MS, samples were kept at room temperature for 2 h.

### 2.5. GC-MS Analysis

A GC (7890; Agilent Technologies, USA) paired with an MS (5975; Agilent) and fitted including an HP-INNOWAX (60 m × 0.25 mm id) capillary column with such a 0.25 µm film thickness was used for the detection of VOCs in raisins and UFAO model reaction, using a previous procedure (Wang et al., 2015). Helium was applied as a carrier gas (1 mL/min), and the SPME (solid-phase microextraction) was injected in splitless mode. The oven temperature was first set at 50 °C for 1 min, then suddenly raised to 220 °C (3 °C/min) and maintained for 5 min at 220 °C. After that, it was progressively increased to 250 °C (5 °C/min) and kept constant for 5 min.

The mass spectra were obtained in the electron impact mode at 230 °C and 70 eV for the source temperature and ionization energy (EI) mode, respectively. After acclimating to a mass range of 20–450 *m*/*z*, full-scan mode and selected ion mode (autotune) were both used for the acquisition. The retention times of n-alkane (C6–C24) were used to determine retention indices in a similar chromatographic pattern. VOCs were identified based on retention indices of known standards, and the mass spectra were matched to the NIST-08 collection. In the absence of a reference standard, a preliminary identification was made using the mass spectrum of the NIST-08 library and retention indices from earlier studies.

### 2.6. Quantification

The quantification method was improved following our prior findings by making necessary modifications [26]. The raisin imitation solution was constructed using the acid and sugar concentrations found in the real raisin solution. In distilled water, a mock solution was prepared with glucose (400 g/L) and tartaric acid (5 g/L), and the pH (4.2) was maintained using a NaOH solution (1 M). For the spike, ethanol (HPLC-standard) was mixed with the known concentration of standard VOCs, which were then diluted into a 15-level using a simulated raisin solution. The raisin supernatant was evaluated in the same way as the standard VOC solutions (15 levels). All aroma compounds had regression coefficients of calibration curves that were over 0.99 in terms of quantification. In the absence of standard compounds, the quantification of compounds was estimated by comparison with those standard curves that hold an identical number of C-atoms or the same functional groups. The amount of detected aroma volatiles was checked by distinguished ion peak areas for 4-methyl-2-pentanol as “internal standard” (1.0018 mg/L).

### 2.7. Statistical Analysis

The volatiles identified from ‘Thompson Seedless’ were statistically analyzed, and the statistical significance of the drying period was evaluated by one-way ANOVA at a significance level of *p* < 0.05 with SPSS software, version 20.0 (IBM corp., Chicago, IL, USA). The heatmaps were created using the Metabo-Analyst 5.0 software (McGill University, Montreal, QC, Canada; http://www.metaboanalyst.ca/) using one-factor analysis for VOC concentrations. To standardize the data, autoscaling was employed (mean-centered and divided by the standard deviation of each variable).

## 3. Results and Discussion

GC-MS was utilized for the characterization and quantification of VOCs; thirty-nine UFAO-derived volatiles was identified (Supplementary Table S1), including 12 aldehydes, 11 alcohols, 7 esters, 5 acids, 2 ketones, 1 furan, and 1 terpene, which are supposed to contribute fruity and roasted flavors [5], generated by enzymatic breakdown and α- and β-oxidation of UFAs in intact/detached fruits. The precursors of the identified volatiles were already reported, but the origin of some compounds such as 1-nonanol, 2-octanol, 2-nonanol, (*Z*)-3-hexen-1-ol, 2-ethyl-1-hexanol, methyl hexadecanoate, ethyl nonanoate, butyrolactone, dodecanoic acid, 2,6-dimethyl-4-heptanone, and 3,4-Dimethyl-2,4,6-octatriene are still unknown.

### 3.1. Free-Form Volatile Compounds Formed during Raisins Drying

Among UFAO-derived compounds, pentanal, hexanal, octanal, (*E*)-2-hexenal, (*E,E*)-2,4-heptadienal, and hexanoic acid were the major bioactive compounds formed in the drying process. In addition, several other compounds such as hexanal, (*E*)-2-hexenal, 1-pentanol, 1-hexanol, ethyl hexanoate, and ethyl octanoate originated from linoleic acids [18,19,27], which were shown to be more concentrated in fresh grapes and showed a decline with the prolonged drying process (Table 1). These findings suggest that these volatile compounds were easily volatilized under the sun-drying process due to the higher potency of light and temperature [5,6,17]. Regardless of these, the content of other compounds from aldehydes such as (*E,E*)-2,4-nonadienal and (*E,E*)-2-octenal produced by linoleic acids [18,19] and alcohols (1-heptanol, 1-octanol and (*E*)-2-octen-1-ol) generated from oleic acids [28,29] were significantly higher on the eighth day of drying. Whereas the acidic compounds such as pentanoic acid, hexanoic acid, and heptanoic acid came from methyl linoleic acid [30], increased with the drying time of ‘Thompson Seedless’ grapes and attained the peak values on the 12th day and then gradually declined in concentration (Table 1). These findings demonstrated that the volatility of ‘Thompson Seedless’ raisins dried in the sun changed significantly as a consequence of moisture changes and sun drying. Some UFAO-derived compounds such as ethyl nonanoate, butyrolactone, and dodecanoic acid were not characterized in fresh grapes; however, they were observed on 4, 4, and 12 days, respectively (Table 1). 2-octanol and 2-nonanol were discovered exclusively in fresh grapes, which may be attributed to their low concentration and ease of volatilization during the drying process.

### 3.2. Optimization of pH for Model Reaction

pH is a key factor for any chemical reaction that plays a vital role in the generation of volatile compounds and also influences their concentration. For optimization of the raisin drying model reaction, the pre-experiment was carried out at three different pHs (4.2, 6.2, and 7). Amongst 27 UFAO-derived compounds, 25, 21, and 21 compounds were found at pH 4.2, 6.2, and 7, respectively (Table 2).

Overall, the concentration of volatiles from the aldehydes, acids, and alcohols group was highest at pH 4.2, whereas the concentration of furan compounds (2-pentyl furan) was utmost at pH 7 (Figure 1) because it is more favorable for the generation of furan compounds [27]. Different raisins varieties were found to contain 1-octen-3-one [10]; however, in a 10-day reaction, it was only produced at 6.2 pH (Table 2). All of the ester compounds (methyl hexadecanoate, ethyl hexanoate, ethyl octanoate, methyl octanoate, ethyl nonanoate, γ-nonalactone, and butyrolactone), and some compounds from acids (1-nonanol, 2-nonanol, 2-ethyl-1-hexanol and (*Z*)-3-Hexen-1-ol), ketones (3-Octen-2-one and 2,6-dimethyl-4-heptanone) and terpene (3,4-dimethyl-2,4,6-octatriene) were found in ‘Thompson Seedless’ during drying. On the other hand, nonanoic acid and n-decanoic acid were not identified in ‘Thompson Seedless’ raisins but were reported in ‘Centennial Seedless’ raisins [9,10] and also quantified in an optimization study (Table 2).

### 3.3. UFAO Model Reaction

In all, 45 VOCs were discovered in UFAO-derived model reactions utilizing the NIST chemistry webbook and Kovats index (Table 3). An authentic reference standard was used to establish the identification of 19 compounds. Among 45 VOCs, eight raisins compounds such as 2-octanol, 2-nonanol, (*Z*)-3-hexen-1-ol, methyl hexadecanoate, methyl octanoate, butyrolactone, dodecanoic acid 3,4-dimethyl-2,4,6-octatriene (Table 1) were not identified in compounds that were produced in the model reaction (Table 3). In our pilot experiment, (*Z*)-3-hexen-1-ol was detected at 120 °C, but it was not discovered in the reaction that was carried out at 60 °C. On the other hand, some compounds such as (*E*)-2-pentenal, (*E*)-2-decanal, 2-undecenal, (*E,E*)-2,4-hexadienal, (*E,E*)-2,4-octadienal, (*E,E*)-2,4-decadienal, 2-heptanol, ethyl hexadecanoate, ethyl decanoate, nonanoic acid, n-decanoic acid, 1-octen-3-one and (*E,E*)-3,5-octadien-2-one were characterized in the model reaction (Table 3) but not found in ‘Thompson Seedless’ during the drying process (Table 1). Among these compounds, ethyl hexadecnoate, ethyl decanoate, nonanoic acid, and n-decanoic acid were reported in free- and bound-form air-dried raisins that came from Crimson Seedless, ‘Thompson Seedless’, and ‘Flame Seedless’ [10], while ethyl heptanoate was found in table grapes [31]. Additionally, Buttery [32] claimed that (*E*)-2-decanal, 2-undecenal, and (*E,E*)-2,4-decadienal were also produced in raisins (Table 3).

#### 3.3.1. Control and Stearic Acid

Only 2,6-dimethyl-4-heptanone was found in control and stearic acid on all reaction days (0, 4, 8, 12, 16, and 20). The 2,6-dimethyl-4-heptanone has been detected in raisins dried by air [10] or sun [6], as well as during storage [5]. The source of 2,6-dimethyl-4-heptanone was not cited in the literature and our outcome indicated that it could not be generated from fatty acids; however, it might be produced due to the heat of the reaction.

#### 3.3.2. Oleic Acid (C18:1)

**Figure 2:**
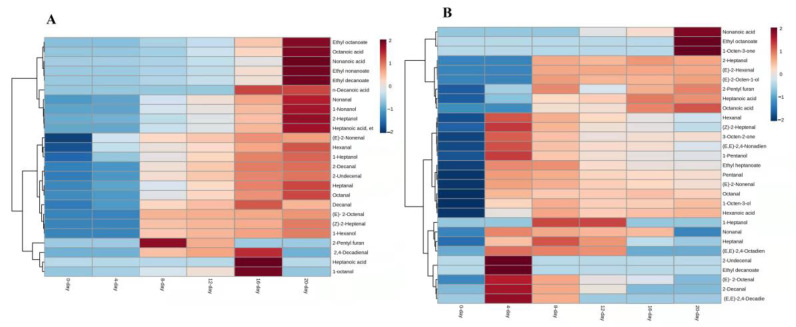
Heatmap visualization of UFAO-derived compounds in model reaction by oleic acid (**A**) and linoleic acid (**B**).

Twenty-five compounds originating from the oleic acid, including 11 aldehydes, 5 esters, 5 alcohols, 3 acids, and 1 furan were enlisted in Table 3. Most of the compounds were generated on the eighth day of the model reaction; however, octanal and nonanal were detected on the day of the reaction, which might be they were easily oxidized (Supplementary Table S2). Heptonic acid was only identified on sixteen-day and its concentration was very low (Figure 2A). As compared to linoleic acid, it was seen in all reaction days with higher content, except 0-day (Supplementary Table S2). These findings indicate that linoleic acids are the main source of heptonic acid.

Overall, the concentration of compounds generated by oleic acid was increased as the reaction time proceeded and highly concentrated at 20 days, except 2-pentyl furan, (*E*,*E*)-2,4-decadienal, heptonic acid and 1-octanol (Figure 2A). The higher amount of 2-pentyl furan, and (*E*,*E*)-2,4-decadienal, heptonic acid and 1-octanol were recorded at 12 and 16 days, respectively (Figure 2A). Among 25 VOCs from oleic acids, some compounds such as octanal, decanal, 1-heptanol, and methyl octanoate were already reported [18,19,28].

#### 3.3.3. Linoleic Acid (C18:2)

In this model reaction, 29 compounds were identified with the reaction of linoleic acid. Only, hexenal, (*E*)-2-heptenal, 1-octen-3-ol, and 3-octen-2-one came from linoleic acid [28] and arachidonic acid [18], respectively, and were found in all reaction days. Furthermore, ethyl octanoate and 1-octen-3-one were merely produced in 20 days of reaction. The (*E,E*)-2,4-octadienal were not produced during the drying of ‘Thompson Seedless’ grapes but it was only generated by the reaction of linoleic acid during 4, 8 and 12 days (Table S2).

The compounds produced by linoleic acid were more concentrated at 4 days and then their content showed a decreasing trend with the time of model reaction. On the other hand, some compounds viz. nonanoic acid, ethyl octanoate, 1-octen-3-one, 2-heptanol, (*E*)-2-hexenal, (*E*)-2-octen-1-ol, 2-pentyl furan, heptanoic acid, and octanoic acids were increased gradually with reaction time and found the highest content on the last day of the reaction (20 days; Figure 2B).

#### 3.3.4. Linolenic Acid (C18:3)

**Figure 3:**
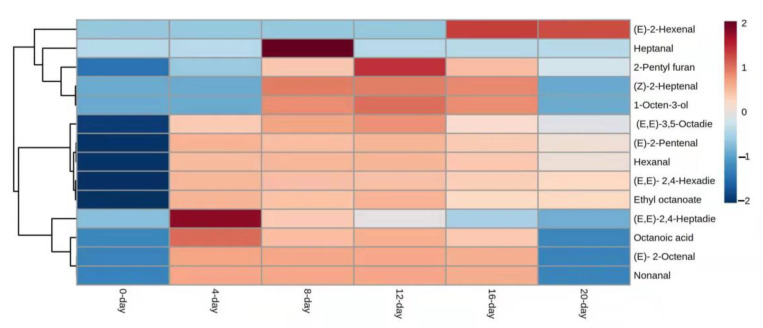
Heatmap visualization of UFAO-derived compounds in model reaction by linolenic acid.

Among other fatty acids, the least number of compounds (14) were characterized in linolenic acid which might be due to the triple bond that needs more energy to produce volatile compounds. Only, (*E,E*)-2,4-heptadienal and (*E,E*)-3,5-Octadien-2-one were produced at the start of reactions, and also were found on other days (4, 8, 12, 16, and 20). Whitfield and Mottram (1992) reported that heptanal was produced by oleic acid and linolenic acid and it was one of the concentrated compounds which was found on the eighth day (Figure 3).

#### 3.3.5. Erucic Acid (C22:1)

In the erucic acid reaction, 27 compounds were generated comprising 11, 6, 5, and 5 coming from aldehydes, esters, alcohols, and acids, respectively. The compounds such as heptanal, octanal, nonanal, 1-heptanol, and decanal originated from oleic acid and linoleic acids [18,19,30] and were identified in all days of the model reaction (Table 3) and ‘Thompson Seedless’ grapes (Table 1), as well. Apart from these, some compounds (ethyl hexanoate, ethyl hexadecnoate, and n-decanoic acid) were only found within 20 days of the reaction (Supplementary Table S2).

**Figure 4:**
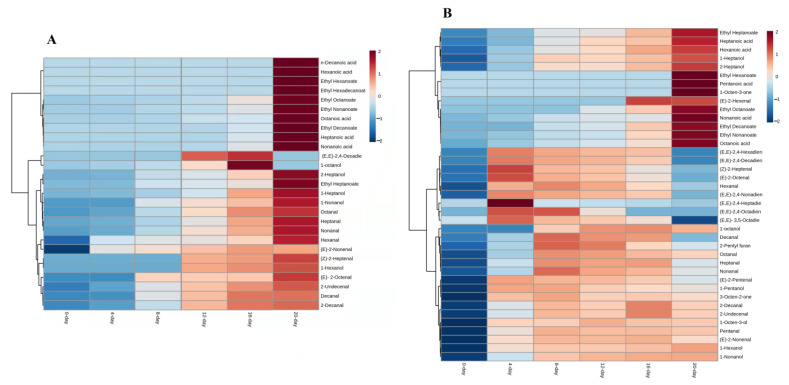
Heatmap visualization of UFAO-derived compounds in model reaction by erucic acid (**A**) and all fatty acid (**B**).

All compounds that were produced by erucic acids were more concentrated at 20 days of reaction (Figure 4A), except 1-octanol and (*E*,*E*)-2,4-decadienal because they were not only found at 20 days (Supplementary Table S2). The concentration of most generated compounds was increased with the time of reaction. The identified ester compounds were higher in concentrations as compared to other identified compounds in erucic acid reactions.

#### 3.3.6. All Fatty Acids

The reaction which contains all fatty acids has produced more volatile compounds (39) than the reaction carried out by single fatty acids. In this reaction, mostly volatiles were produced at 4 days of reaction while pentanoic acid, ethyl hexanoate, and 1-octen-3-one were found at 20 days of reaction. Pentanoic acid was the only compound identified in all fatty acids on the last day of the reaction (Supplementary Table S2). Among 39 volatiles, heptanal, octanal, nonanal, (*E*)-2-heptenal, (*E*)-2-octanal, and 1-octen-3-ol were those compounds identified in all the reaction days of the experiment.

The ester compounds were produced from oleic acid, linoleic acid, and linolenic acid. These compounds were also identified in fatty acid mixture reactions and showed an increase in their concentration with the time of reaction [33,34]. Some aldehydes compounds such as (*E,E*)-2,4-decadienal, (*E,E*)-2,4-nonadienal, (*E,E*)-2,4-octadienal, (*E*)-2-octanal, (*E,E*)-2,4-heptadienal, (*E*)-2-heptenal, (*E,E*)-2,4-hexadienal, and hexanal were more abundant at the fourth day of reaction while pentanal, heptanal, octanal, nonanal, decanal, (*E*)-2-nonenal, (*E*)-2-decanal and (*E*)-2-undecanal were higher on the eighth day and then went down as the reaction time proceed (Figure 4B).

## 4. Conclusions

This study comprises two parts; (1) identification of UFAO-derived compounds during drying of ‘Thompson Seedless’, and (2) designing a model reaction to identify the raisin volatiles using different fatty acids (stearic acid, oleic acid, linoleic acid, linolenic acid, erucic acid, and mixture of all) at 60 °C and 0, 4, 8, 12, 16, and 20 days. GC-MS quantification demonstrated 39 compounds identified from dried ‘Thompson Seedless’, including aldehydes, alcohols, esters, and acids. Regarding the model reaction, firstly a pre-experiment was designed to optimize the pH for raisins, and a pH of 4.2 was selected for the model reaction based on the higher number of volatiles and their concentration. A total of 46 compounds were identified in the model reaction. Among these, 14, 25, 27, 29, and 39 were produced from linolenic acids, oleic acids, erucic acids, linoleic acids, and all fatty acids, respectively. Pentanoic acid, (*E,E*)-2,4-octadienal, and *n*-decanoic acid were only quantified in all fatty acids, linoleic acid, and erucic acid, respectively. Some compounds such as pentanal, (*E,E*)-2,4-nonadienal, 1-pentanol, (*E*)-2-octen-1-ol, 1-octen-3-one and 3-octen-2-one were found in linoleic acid, while (*E*)-2-pentenal, (*E,E*)-2,4-hexadienal, (*E,E*)-2,4-octadienal and (*E,E*)-3,5-octadien-2-one in linolenic acid were also identified in the interaction of all fatty acids. There were some compounds such as 2-octanol, 2-nonanol, (*Z*)-3-hexen-1-ol, dodecanoic acid, butyrolactone, and 3,4-dimethyl-2,4,6-octatriene found in ‘Thompson Seedless’ during drying but could not be identified in the model reaction. Further research studies are required to identify the missing compounds, which might be explored by the interactions of sugars with amino acids.

## Figures and Tables

**Table 1:** The concentration (µg/L) of UNFO-derived compounds in ‘Thompson Seedless’ at different days of drying.

S.No	RI	Compound Name	ID	0-Day	4-Day	8-Days	12-Days	16-Days	20-Day
1	975	Pentanal	2	147.6 ± 20.86 **c**	198.8 ± 9.24 **b**	223.1 ± 19.20 **a**	113.5 ± 6.61 **d**	93.3 ± 11.44 **e**	88.9 ± 2.88 **e**
2	1066	Hexanal	1	5802.8 ± 193.9 **a**	2036.6 ± 67.3 **b**	1491.3 ± 126.3 **c**	594.9 ± 39.94 **d**	559.2 ± 18.45 **e**	534.4 ± 14.85 **f**
3	1132	3,4-Dimethyl-2,4,6-octatriene	2	35.44 ± 3.65 **a**	15.54 ± 0.72 **b**	10.53 ± 0.90 **c**	7.88 ± 0.16 **d**	9.68 ± 0.46 **cd**	16.76 ± 0.82 **b**
4	1167	2,6-Dimethyl-4-heptanone	2	139.93 ± 2.81 **a**	19.68 ± 2.84 **b**	5.26 ± 0.54 **d**	1.91 ± 0.06 **e**	0.87 ± 0.14 **f**	7.53 ± 1.14 **c**
5	1178	Heptanal	2	62.8 ± 6.18 **b**	68.8 ± 5.20 **b**	76.5 ± 8.82 **a**	42.6 ± 2.90 **c**	41.6 ± 2.44 **c**	45.4 ± 3.52 **c**
6	1205	1-Pentanol	2	87.752 ± 16.38 **a**	79.714 ± 1.853 **ab**	69.93 ± 1.752 **c**	35.408 ± 3.42 **d**	16.386 ± 0.12 **e**	9.42 ± 0.297 **f**
7	1217	(*E*)-2-Hexenal	2	2892.9 ± 81.9 **a**	505.1 ± 26.71 **b**	104.1 ± 6.42 **c**	31.84 ± 2.70 **d**	16.39 ± 0.80 **e**	13.36 ± 0.91 **f**
8	1224	2-Pentyl furan	2	34.09 ± 2.66 **f**	65.92 ± 4.30 **e**	241.47 ± 21.31 **a**	220.56 ± 18.62 **ab**	159.53 ± 15.84 **c**	116.34 ± 8.02 **d**
9	1227	Ethyl hexanoate	2	41.105 ± 2.923 **a**	18.01 ± 0.92 **b**	11.75 ± 0.07 **c**	8.14 ± 0.05 **d**	7.59 ± 0.91 **e**	7.68 ± 0.58 **e**
10	1292	Octanal	2	353.2 ± 84.02 **a**	147.98 ± 24.162 **b**	69.3 ± 2.86 **c**	66.2 ± 1.48 **c**	61.2 ± 1.77 **c**	52.5 ± 1.05 **d**
11	1325	(*E*)-2-Heptenal	2	23.32 ± 9.27 **e**	50.94 ± 22.67 **d**	365.6 ± 91.98 **a**	121.8 ± 4.24 **b**	78.1 ± 3.47 **c**	49.08 ± 2.86 **d**
12	1349	1-Hexanol	1	491.7 ± 43.57 **a**	267.5 ± 53.79 **b**	153.6 ± 11.38 **c**	76.2 ± 13.85 **d**	51.8 ± 13.83 **e**	40.5 ± 7.17 **f**
13	1378	Ethyl octanoate	2	0.68 ± 0.042 **a**	0.57 ± 0.043 **b**	0.49 ± 0.094 **c**	0.44 ± 0.13 **d**	0.34 ± 0.044 **e**	0.34 ± 0.027 **e**
14	1393	Nonanal	1	5.32 ± 0.77 **a**	3.917 ± 0.52 **b**	3.46 ± 0.76 **b**	2.391 ± 0.22 **c**	2.15 ± 0.14 **c**	1.75 ± 0.18 **d**
15	1395	(*Z*)-3-Hexen-1-ol	2	188.5 ± 9.96 **c**	271.7 ± 5.84 **b**	304.6 ± 27.02 **a**	170.2 ± 13.01 **c**	141.9 ± 19.81 **d**	104.5 ± 7.32 **e**
16	1411	2-Octanol	2	0.893 ± 0.42 **a**	NF	NF	NF	NF	NF
17	1416	3-Octen-2-one	2	3.93 ± 0.59 **e**	4.13 ± 0.28 **e**	34.85 ± 4.02 **a**	17.45 ± 0.54 **b**	11.34 ± 0.144 **c**	10.40 ± 0.358 **cd**
18	1434	(*E*)-2-Octenal	2	24.90 ± 0.65 **d**	36.41 ± 2.14 **b**	81.61 ± 11.56 **a**	36.82 ± 2.84 **b**	30.31 ± 0.68 **c**	19.98 ± 0.18 **e**
19	1449	1-Octen-3-ol	1	13.95 ± 1.16 **e**	43.76 ± 3.03 **c**	202.04 ± 29.17 **a**	52.03 ± 3.73 **b**	40.64 ± 1.33 **c**	34.18 ± 0.71 **d**
20	1453	1-Heptanol	2	5.56 ± 0.62 **c**	9.18 ± 0.48 **b**	15.60 ± 2.81 **a**	9.23 ± 0.89 **b**	5.19 ± 0.63 **c**	5.46 ± 0.282 **d**
21	1487	2-Ethyl-1-hexanol	1	10.58 ± 0.53 **b**	16.84 ± 0.56 **a**	11.63 ± 1.34 **b**	4.40 ± 0.73 **c**	2.06 ± 0.29 **d**	1.77 ± 0.04 **e**
22	1488	2-Nonanol	2	1.59 ± 0.12 **a**	NF	NF	NF	NF	NF
23	1497	(*E,E*)-2,4-Heptadienal	2	2266.1 ± 135.4 **a**	858.6 ± 114.3 **b**	238.3 ± 5.9 **c**	160.1 ± 8.8 **d**	107.9 ± 5.16 **e**	113.1 ± 3.82 **e**
24	1501	Decanal	1	6.76 ± 0.17 **a**	4.92 ± 0.66 **b**	4.74 ± 0.13 **b**	4.19 ± 0.27 **bc**	3.31 ± 0.17 **c**	2.92 ± 0.66 **cd**
25	1539	(*E*)-2-Nonenal	2	10.79 ± 1.75 **d**	21.10 ± 0.67 **a**	19.57 ± 0.46 **b**	16.38 ± 2.20 **c**	11.66 ± 2.40 **d**	14.50 ± 1.59 **c**
26	1555	1-Octanol	1	4.31 ± 0.44 **cd**	4.07 ± 0.35 **cd**	12.51 ± 1.28 **a**	6.67 ± 0.19 **b**	4.98 ± 0.16 **c**	4.67 ± 0.05 **c**
27	1570	Methyl octanoate	2	5.75 ± 0.21 **a**	4.47 ± 0.30 **b**	4.72 ± 0.69 **b**	2.31 ± 0.22 **c**	2.51 ± 0.33 **c**	2.44 ± 0.27 **c**
28	1570	Ethyl nonanoate	1	NF	0.31 ± 0.03 **a**	0.25 ± 0.01 **b**	0.181 ± 0.008 **c**	0.184 ± 0.016 **c**	0.185 ± 0.023 **c**
29	1614	(*E*)-2-Octen-1-ol	2	3.29 ± 0.267 **d**	5.876 ± 0.795 **c**	30.403 ± 3.094 **a**	12.281 ± 1.54 **b**	6.219 ± 1.253 **c**	5.687 ± 0.132 **c**
30	1636	Butyrolactone	2	NF	3.31 ± 0.12 **c**	9.70 ± 1.32 **a**	7.34 ± 0.20 **b**	7.19 ± 0.29 **b**	7.09 ± 0.27 **b**
31	1657	1-Nonanol	1	1.37 ± 0.105 **bc**	1.53 ± 0.059 **b**	2.40 ± 0.209 **a**	1.49 ± 0.114 **b**	1.14 ± 0.022 **cd**	1.05 ± 0.08 **d**
32	1740	Pentanoic acid	2	71.409 ± 4.741 **e**	95.02 ± 5.60 **d**	194.4 ± 14.15 **b**	256.3 ± 15.81 **a**	205.7 ± 7.49 **b**	181.6 ± 6.67 **bc**
33	1750	(*E,E*)-2,4-Nonadienal	1	266.4 ± 35.7 **c**	268.7 ± 39.5 **c**	673.8 ± 204.7 **a**	621.2 ± 23.45 **ab**	477.1 ± 72.70 **b**	297.9 ± 9.41 **c**
34	1847	Hexanoic acid	1	284.1 ± 24.71 **d**	393.6 ± 8.35 **c**	468.8 ± 74.12 **b**	633.6 ± 29.01 **a**	498.0 ± 22.30 **b**	390.7 ± 7.68 **c**
35	1953	Heptanoic acid	1	1.52 ± 0.33 **e**	2.20 ± 0.12 **d**	5.06 ± 0.66 **c**	7.23 ± 0.67 **a**	5.96 ± 0.29 **b**	5.02 ± 0.23 **c**
36	2035	γ-Nonalactone	2	2.49 ± 0.12 **f**	5.40 ± 0.33 **c**	10.84 ± 1.17 **a**	9.36 ± 1.29 **ab**	4.35 ± 0.27 **d**	3.99 ± 0.13 **e**
37	2060	Octanoic acid	1	1.37 ± 0.20 **e**	1.85 ± 0.20 **d**	4.86 ± 0.41 **bc**	5.18 ± 0.37 **b**	5.42 ± 0.46 **b**	7.26 ± 0.81 **a**
38	2163	Methyl hexadecanoate	2	0.392 ± 0.009 **a**	0.173 ± 0.014 **b**	0.132 ± 0.023 **c**	0.079 ± 0.002 **d**	0.072 ± 0.009 **d**	0.086 ± 0.004 **d**
39	2484	Dodecanoic acid	2	NF	NF	NF	14.15 ± 1.13 **a**	12.77 ± 2.12 **b**	8.52 ± 1.65 **c**

Retention Indices (RI): Kovats retention indices were calculated based on a *n*-alkane series (C6–C24) on the poly (ethylene glycol) Retention Indices (RI): Kovats retention indices were calculated based on a *n*-alkane series (C6–C24) on the poly (ethylene glycol) (PEG) column under the same chromatographic conditions. Identification method (ID-M): 1, identified, mass spectrum and RI were in accordance with standards; 2, tentatively identified, mass spectrum matched in the standard NIST 2008 library and RI matched with NIST Standard Reference Database (NIST Chemistry WebBook). NF = Not found; Mean ± standard deviation (*n* = 3) of the same compounds followed by different letters are significantly different (*p* < 0.05), the free volatiles were compared separately.

**Table 2:** The concentration (µg/L) of identified volatile compounds in Model reaction at different pH.

			10-Days			20-Days		
S.No	Compounds		4.2	6.2	7.0	4.2	6.2	7.0
1	Pentanal	Aldehyde	3060.5 ± 193.6	1521.3 ± 59.3	835.05 ± 25.2	3283.5 ± 110.9	1834.2 ± 111.3	1632.0 ± 62.7
2	Hexanal	Aldehyde	20,851.8 ± 361.2	15,511.1 ± 576.9	9977.3 ± 233.9	16,535.1 ± 647.2	14,231.2 ± 326.1	14,814.0 ± 46.8
3	Heptanal	Aldehyde	1644.1 ± 72.5	708.3 ± 65.4	510.4 ± 22.1	1499.0 ± 76.8	976.1 ± 113.8	305.4 ± 70.0
4	Octanal	Aldehyde	4377.1 ± 35.0	1289.3 ± 76.4	909.8 ± 52.8	4309.4 ± 36.1	2048.5 ± 103.4	1577.1 ± 104.6
5	Nonanal	Aldehyde	11,677.7 ± 105.4	1993.2 ± 88.2	1333.1 ± 32.5	7830.0 ± 62.8	1704.6 ± 86.1	1606.4 ± 49.6
6	Decanal	Aldehyde	865.0 ± 4.2	NF	1592.2 ± 118.4	546.3 ± 44.8	116.1 ± 7.0	55.9 ± 7.8
7	(*E*)-2-Hexenal	Aldehyde	21,025.7 ± 552.4	70,294.4 ± 3647.2	NF	19,286.5 ± 260.2	22,939.2 ± 320.3	NF
8	(*E*)-2-Heptenal	Aldehyde	3822.6 ± 69.6	2006.0 ± 50.1	986.1 ± 75.7	2753.5 ± 96.7	642.8 ± 38.4	458.3 ± 46.0
9	(*E*)-2-Octenal	Aldehyde	8388.9 ± 189.6	2161.1 ± 85.6	2580.6 ± 43.3	4583.4 ± 126.4	522.3 ± 11.1	439.9 ± 26.6
10	(*E*)-2-Nonenal	Aldehyde	773.3 ± 20.6	1275.4 ± 27.7	7582.4 ± 401.5	537.8 ± 7.8	367.6 ± 45.2	245.0 ± 31.0
11	(*E,E*)-2,4-Heptadienal	Aldehyde	6032.3 ± 143.0	NF	NF	2745. ± 149.1	1682.9 ± 91.2	1535.6 ± 176.0
12	(*E,E*)-2,4-Nonadienal	Aldehyde	875.8 ± 63.3	609.5 ± 6.6	113.1 ± 2.0	393.2 ± 2.4	209.8 ± 7.0	232.9 ± 13.8
13	1-Pentanol	Alcohol	34,425.3 ± 1551.2	20,350.9 ± 69.4	11,424.4 ± 319.7	26,752.6 ± 1114.4	25,294.4 ± 492.0	22,243.6 ± 62.1
14	1-Hexanol	Alcohol	1487.4 ± 70.7	NF	NF	NF	NF	NF
15	1-Heptanol	Alcohol	694.3 ± 11.9	514.9 ± 64.1	NF	797.1 ± 11.8	929.1 ± 10.1	588.6 ± 10.1
16	1-Octanol	Alcohol	3189.3 ± 114.5	1984.4 ± 47.6	NF	3028.0 ± 56.1	1795.8 ± 52.1	1544.1 ± 47.3
17	1-Octen-3-ol	Alcohol	1768.1 ± 27.5	1747.6 ± 46.7	NF	1647.7 ± 61.4	1434.3 ± 83.3	1132.3 ± 2.2
18	2-Octanol	Alcohol	NF	NF	204.9 ± 10.0	NF	NF	NF
19	(*E*)-2-Octen-1-ol	Alcohol	209.72 ± 2.2	NF	NF	193.6 ± 4.3	NF	NF
20	Pentanoic acid	Acid	3176.5 ± 101.0	NF	31,214.3 ± 219.8	NF	NF	NF
21	Hexanoic acid	Acid	5821.4 ± 74.6	188.3 ± 37.8	NF	4775.0 ± 87.0	586.2 ± 1.7	181.7 ± 13.7
22	Heptanoic acid	Acid	3368.9 ± 75.1	NF	NF	3064.4 ± 7.9	NF	NF
23	Octanoic acid	Acid	3888.9 ± 79.7	289.5 ± 7.4	363.0 ± 28.2	5299.7 ± 436.7	NF	NF
24	Nonanoic acid	Acid	4377.1 ± 35.0	1389.3 ± 76.4	909.8 ± 52.8	4409.4 ± 177.6	2048.5 ± 103.4	1527.1 ± 175.3
25	*n*-Decanoic acid	Acid	NF	NF	NF	193.1 ± 7.07	NF	NF
26	2-Pentyl furan	Furan	27,093.8 ± 388.4	19,343.9 ± 393.4	20,022.4 ± 95.7	25,569.7 ± 274.1	7054.2 ± 142.6	15,284.7 ± 3161.8
27	1-Octen-3-one	Ketone	NF	785.4 ± 29.3	NF	NF	NF	NF

**Table 3:** The UFAO-derived compounds produced their respective fatty acids in raisin-like model reaction.

S.No	CAS #	Final Compound Name	Source	Aromatic Series	Precursor
	Unsaturated Fatty Acid				
1	110-62-3	Pentanal	Aldehyde	Fat, Green ^f^	Linoleic acid, Mixture
2	66-25-1	Hexanal	Aldehyde	Green ^f^	Oleic acid, Linoleic acid, Linolenic acid, Erucic acid, Mixture
3	111-71-7	Heptanal	Aldehyde	Dry fish, solvent, smoky ^f^	Oleic acid, Linoleic acid, Erucic acid, Mixture
4	124-13-0	Octanal	Aldehyde	Honey, Green, Fatty ^f^	Oleic acid, Linoleic acid, Erucic acid, Mixture
5	124-19-6	Nonanal	Aldehyde	Green, Fruity ^f^	Oleic acid, Linoleic acid, Linolenic acid, Erucic acid, Mixture
6	112-31-2	Decanal	Aldehyde	Sweet, citrus, green ^d^	Oleic acid, Erucic acid, Mixture
7	1576-87-0	(*E*)-2-Pentenal	Aldehyde	Apple, green ^b^	Linolenic acid, Mixture
8	505-57-7	(*E*)-2-Hexenal	Aldehyde	Green ^f^	Linoleic acid, Linolenic acid, Mixture
9	2463-63-0	(*E*)-2-Heptenal	Aldehyde	Fatty, soapy, tallow ^f^	Oleic acid, Linoleic acid, Linolenic acid, Erucic acid, Mixture
10	2548-87-0	(*E*)-2-Octenal	Aldehyde	Green, fatty, nut ^f^	Oleic acid, Linoleic acid, Linolenic acid, Erucic acid, Mixture
11	18829-56-6	(*E*)-2-Nonenal	Aldehyde	Green, fat ^a^	Oleic acid, Linoleic acid, Erucic acid, Mixture
12	3913-81-3	(*E*)-2-Decanal	Aldehyde	Green, fat	Oleic acid, Linoleic acid, Erucic acid, Mixture
13	1337-83-3	2-Undecenal	Aldehyde	Sweet ^a^	Oleic acid, Erucic acid, Mixture
14	142-83-6	(*E,E*)-2,4-Hexadienal	Aldehyde	Green ^a^	Linolenic acid, Mixture
15	4313--03-5	(*E,E*)-2,4-Heptadienal	Aldehyde	Fatty, hay ^b^	Linolenic acid, Mixture
16	30361-28-5	(*E,E*)-2,4-Octadienal	Aldehyde	Green, cucumber ^a^	Linoleic acid
17	5910-87-2	(*E,E*)-2,4-Nonadienal	Aldehyde	Fatty, oily ^b^	Linoleic acid, Mixture
18	25152-84-5	(*E,E*)-2,4-Decadienal	Aldehyde	Seaweed ^a^	Oleic acid, Erucic acid, Mixture
19	74-41-0	1-Pentanol	Alcohol	Balsamic, almond ^c^	Linoleic acid, Mixture
20	111-27-3	1-Hexanol	Alcohol	green ^b^	Oleic acid, Erucic acid, Mixture
21	111-70-6	1-Heptanol	Alcohol	Grape, sweet ^c^	Oleic acid, Linoleic acid, Erucic acid, Mixture
22	111-87-5	1-Octanol	Alcohol	Citrus, rose ^c^	Linoleic acid, Erucic acid, Mixture
23	143-08-8	1-Nonanol	Alcohol	Floral ^b^	Oleic acid, Erucic acid, Mixture
24	3391-86-4	1-Octen-3-ol	Alcohol	Mushroom, fruity ^b,f^	Linolenic acid, Mixture
25	104-76-7	2-Ethyl-1-hexanol	Alcohol	Floral, sweet fruity ^c^	Linolenic acid
26	543-49-7	2-Heptanol	Alcohol	Herbaceous, lemon	Oleic acid, Linoleic acid, Erucic acid, Mixture
27	18409-17-1	(*E*)-2-Octen-1-ol	Alcohol	Fatty, rancid ^b^	Linoleic acid, Mixture
28	628-97-7	Ethyl Hexadecanoate	Ester	Wax ^a^	Oleic acid, Erucic acid
29	123-66-0	Ethyl Hexanoate	Ester	Fruity, apple like ^a,f^	Erucic acid, Mixture
30	106-30-9	Ethyl Heptanoate	Ester	Fruit ^a^	Oleic acid, Linoleic acid, Erucic acid, Mixture
31	106-32-1	Ethyl Octanoate	Ester	Fruity, citrus like ^a^	Oleic acid, Linoleic acid, Linolenic acid, Erucic acid, Mixture
32	123-29-5	Ethyl Nonanoate	Ester	Fruity, floral ^b^	Oleic acid, Linoleic acid, Erucic acid, Mixture
33	110-38-3	Ethyl Decanoate	Ester	Grape ^a^	Oleic acid, Linoleic acid, Erucic acid, Mixture
34	104-61-0	γ-Nonalactone	Ester	Coconut, peach ^a^	Oleic acid, Linoleic acid, Mixture
35	109-52-4	Pentanoic acid	Acid	Sweet ^a^	Mixture
36	142-62-1	Hexanoic acid	Acid	Rancid, Chees, Fatty ^f^	Linoleic acid, Erucic acid, Mixture
37	111-14-8	Heptanoic acid	Acid	Sweety, cheesy ^e^	Oleic acid, Linoleic acid, Erucic acid, Mixture
38	124-07-2	Octanoic acid	Acid	Rancid, Chees, Fatty ^f^	Oleic acid, Linoleic acid, Linolenic acid, Erucic acid, Mixture
39	67762-36-1	Nonanoic acid	Acid	Green, fat ^a^	Oleic acid, Linoleic acid, Erucic acid, Mixture
40	334-48-5	*n*-Decanoic acid	Acid	Raincid ^a^	Erucic acid
41	4312-99-6	1-Octen-3-one	Ketone	Mushroom, metal ^a^	Linoleic acid, Mixture
42	1669-44-9	3-Octen-2-one	Ketone	Green, fruity ^b^	Linoleic acid, Mixture
43	108-83-8	2,6-Dimethyl-4-heptanone	Ketone	NF	Control, Stearic acid, Oleic acid, Linoleic acid, Linolenic acid, Erucic acid, Mixture
44	3777-69-3	2-Pentyl furan	Furan	Fruity, green, sweet ^f^	Oleic acid, Linoleic acid, Linolenic acid, Mixture
45	30086-02-3	(*E,E*)-3,5-Octadien-2-one		NF	Linolenic acid, Mixture

Aroma descriptors were obtained from “Flavornet and human odor space” (^a^ http://www.flavornet.org/flavornet.html, accessed on 19 September 2019), the LRI and odor database (^b^ http://www.odour.org.uk/odour/ index.html, accessed on 23 June 2021) and from reported literature (^c^ Jiang and Zhang [33]; ^d^ Wang et al. [6]; ^e^ Welke et al. [34]; ^f^ Wu et al. [31].

## Data Availability

Data is contained within the article or supplementary material.

## Supplementary Materials

The following supporting information can be downloaded at: https://www.mdpi.com/article/10.3390/foods12030428/s1. Figure S1: Effect of sun-drying method on the moisture loss of ’Thompson Seedless’ grape. Table S1: Descriptions of identified UFAO-derived compounds during drying in ‘Thompson Seedless’ grape. Table S2: Formation of UFAO compounds by the reaction of different fatty acids in a raisin-like Model Reaction.
