# Supplementary material for: Interrogating Raisin Associated Unsaturated Fatty Acid Derived Volatile Compounds Using HS–SPME with GC–MS
Source: Foods. 2023 Jan 17;12(3):428. doi: 10.3390/foods12030428 (PMC9914242; doi:10.3390/foods12030428)
Supplement: Supplementary file 1 [file foods-12-00428-s001.zip › foods-2152242-supplementary/Supplementary Table S1.pdf]

Table S1: Descriptions of identified UFAO-derived compounds during drying in Thompson seedless grape.

| S.No | CAS # | RI | Final Compound Name | ID-M | Ion (m/z) | Source | OTV (μg/L) | Aromatic series | Precursor |
|---|---|---|---|---|---|---|---|---|---|
| | **Unsaturated Fatty Acid** | | | | | | | | |
| 1 | 110-62-3 | 975 | Pentanal | 2 | 44 | Aldehyde | 12[1,5] | Fat, Green [g] | Linoleic acid, Arachidonic acid [E] |
| 2 | 66-25-1 | 1066 | Hexanal | 1 | 44 | Aldehyde | 4.53[5] | Green [g] | Linoleic acid [E] |
| 3 | 111-71-7 | 1178 | Heptanal | 2 | 44 | Aldehyde | 3[1,5] | Dry fish, solvent, smoky [g] | Linoleic acid [D,E], Oleic acid [E] |
| 4 | 124-13-0 | 1292 | Octanal | 1 | 43 | Aldehyde | 0.7[1,5] | Honey, Green, Fatty [g] | Oleic acid |
| 5 | 124-19-6 | 1393 | Nonanal | 1 | 57 | Aldehyde | 1[1,5] | Green, Fruity [g] | Linoleic acid [B] |
| 6 | 112-31-2 | 1501 | Decanal | 1 | 43 | Aldehyde | 0.1[1] | Sweet, citrus, green [e] | Oleic acid [D,E] |
| 7 | 505-57-7 | 1217 | (*E*)-2-Hexenal | 1 | 41 | Aldehyde | 17[1,5] | Green [g] | Linoleic acid [D,E] |
| 8 | 2463-63-0 | 1325 | (*E*)-2-Heptenal | 2 | 41 | Aldehyde | 13[1,5] | Fatty, soapy, tallow [g] | Linoleic acid [D,E] |
| 9 | 2548-87-0 | 1434 | (*E*)-2-Octenal | 2 | 41 | Aldehyde | 3[1] | Green, fatty, nut [g] | Linoleic acid, Linolenic acid [A, E] |
| 10 | 18829-56-6 | 1539 | (*E*)-2-Nonenal | 2 | 43 | Aldehyde | 0.08[1] | Green, fat [a] | Linoleic acid [D,E] |
| 11 | 4313--03-5 | 1497 | (*E*,*E*)-2,4-Heptadienal | 2 | 81 | Aldehyde | 49[2] | Fatty, hay [b] | Linolenic acid [E] |
| 12 | 5910-87-2 | 1750 | (*E*,*E*)-2,4-Nonadienal | 2 | 81 | Aldehyde | 0.09[1] | Fatty, oily [b] | Linoleic acid [D,E] |
| 13 | 74-41-0 | 1205 | 1-Pentanol | 2 | 52 | Alcohol | 4000[1] | Balsamic, almond [c] | Methyl linoleic acid [C] |
| 14 | 111-27-3 | 1349 | 1-Hexanol | 2 | 56 | Alcohol | 2500[1] | green [b] | Methyl linoleic acid [C] |
| 15 | 111-70-6 | 1453 | 1-Heptanol | 2 | 70 | Alcohol | 1000[1,3] | Grape, sweet [c] | Oleic acid [A] |
| 16 | 111-87-5 | 1555 | 1-Octanol | 1 | 56 | Alcohol | 130[1,2] | Citrus, rose [c] | Methyl Oleate [B] |
| 17 | 143-08-8 | 1657 | 1-Nonanol | 1 | 56 | Alcohol | 50[1] | Floral [b] | NF |
| 18 | 3391-86-4 | 1449 | 1-Octen-3-ol | 1 | 57 | Alcohol | 3[1,5] | Mushroom, fruity [b, g] | Arachidonic acid [D], Linoleic acid [A] |
| 19 | 104-76-7 | 1487 | 2-Ethyl-1-hexanol | 1 | 57 | Alcohol | 270000[1] | Floral, sweet fruity [c] | NF |
| 20 | 123-96-6 | 1411 | 2-Octanol | 2 | 45 | Alcohol | NF | Mushroom, fat [a] | NF |
| 21 | 18409-17-1 | 1614 | (*E*)-2-Octen-1-ol | 2 | 57 | Alcohol | 18[3] | Fatty, rancid [b] | Oleic acid [A] |
| 22 | 628-99-9 | 1488 | 2-Nonanol | 2 | 45 | Alcohol | 58[2] | Fruity, green [d, f] | NF |
| 23 | 928-96-1 | 1395 | (*Z*)-3-Hexen-1-ol | 2 | 67 | Alcohol | 100[1,3] | Fruity, green [b] | NF |
| 24 | 112-39-0 | 2163 | Methyl hexadecanoate | 2 | 74 | Ester | NF | NF | NF |
| 25 | 123-66-0 | 1227 | Ethyl hexanoate | 1 | 88 | Ester | 1[1,5] | Fruity, apple like [a, g] | Linoleic acid [F] |
| 26 | 106-32-1 | 1432 | Ethyl octanoate | 2 | 74 | Ester | 194[1] | Fruity, citrus like [a] | Linoleic acid, Linolenic acid [F] |
| 27 | 111-11-5 | 1417 | Methyl octanoate | 2 | 88 | Ester | 200[1] | Fruity, floral [b] | Oleic acid [A] |
| 28 | 123-29-5 | 1570 | Ethyl nonanoate | 1 | 88 | Ester | 377[1] | Fruity, floral [b] | NF |
| 29 | 104-61-0 | 2035 | γ-Nonalactone | 2 | 85 | Ester | 30[4] | Coconut, peach [a] | Linoleic acid [F] |
| 30 | 96-84-0 | 1636 | Butyrolactone | 2 | 42 | Ester | NF | Caramel, sweet [a] | NF |
| 31 | 109-52-4 | 1740 | Pentanoic acid | 2 | 60 | Acid | 3000[1] | Sweet | Methyl Linoleic acid [C] |
| 32 | 142-62-1 | 1847 | Hexanoic acid | 1 | 60 | Acid | 3000[1,5] | Raincid, Chees, Fatty [g] | Methyl Linoleic acid [C] |
| 33 | 111-14-8 | 1953 | Heptanoic acid | 1 | 60 | Acid | 3000[1] | Sweety, cheesy [f] | Methyl Linoleic acid [C] |

| | | | | | | | | | |
|---|---|---|---|---|---|---|---|---|---|
| 34 | 124-07-2 | 2060 | Octanoic acid | 1 | 60 | Acid | 3000[1,5] | Raincid, Chees, Fatty [g] | Methyl Linoleic acid [C] |
| 35 | 143-07-7 | 2484 | Dodecanoic acid | 2 | 73 | Acid | 10000[1] | Dry, metallic [f] | NF |
| 36 | 1669-44-9 | 1416 | 3-Octen-2-one | 2 | 55 | Ketone | NF | Green, fruity [b] | Arachidonic acid [E] |
| 37 | 108-83-8 | 1167 | 2,6-Dimethyl-4-heptanone | 2 | 57 | Ketone | NF | NF | NF |
| 38 | 3777-69-3 | 1224 | 2-Pentyl furan | 2 | 81 | Furan | 6[1,5] | Fruity, green, sweet [g] | Linoleic acid [B,E] |
| 39 | 57396-75-5 | 1132 | 3,4-Dimethyl-2,4,6-octatriene | 2 | 121 | Terpene | NF | NF | NF |

OTV (Odor Threshold values; Parts per Billion, µg/L) in water were reported by: ([1] Leffingwell & Associates, 2004; [2] Nan et al., 2013; [3] Qian & Wang, 2005; [4] Wang et al., 2017; [5] Wu et al., 2016). Aroma descriptors were obtained from "Flavornet and human odor space" ([a] http://www.flavornet.org/flavornet.html), the LRI and odor database ([b] http://www.odour.org.uk/odour/ index.html) and from reported literature ([c] Jiang & Zhang, 2010; [d] Qian & Wang, 2005; [e] Wang et al., 2017; [f] Welke et al., 2014; [g] Wu et al., 2016). Reported the precursor (fatty acids) of their respected volatile compounds ([A] Frankel 1980; [B] Frankel, Neff, and Edward S. 1981; [C] Horvat et al. 1968; [D] Meeting, Ho, and Hartman 1994; [E] Whitfield and Mottram 1992; [F] Belitz, Garcia and Wayne, 2004); ("NF" indicated not find. Identification method (ID): 1) identified, mass spectrum and RI were following standards; 2) tentatively identified, mass spectrum matched in the standard NIST 2008 library and RI matched with NIST Standard Reference Database (NIST Chemistry WebBook). Kovats retention indices (RI) were calculated based on an n-alkane series (C6–C24) on the poly (ethylene glycol) (PEG) column under the same chromatographic conditions.
