# Supplementary figures and images for: Interrogating Raisin Associated Unsaturated Fatty Acid Derived Volatile Compounds Using HS–SPME with GC–MS
Source: Foods. 2023 Jan 17;12(3):428. doi: 10.3390/foods12030428 (PMC9914242; doi:10.3390/foods12030428)

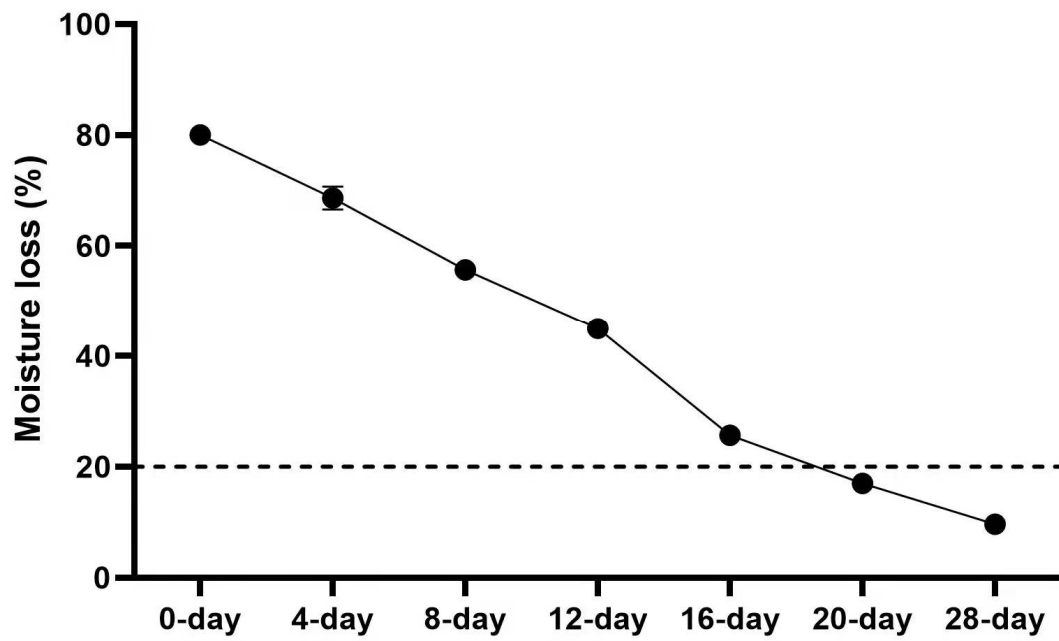

**Figure S1:** Effect of sun-drying method on the moisture loss of; Thompson Seedless' grape.

Supplement: Supplementary file 1 [file foods-12-00428-s001.zip › foods-2152242-supplementary/Supplementary Figure S1.pdf]
